# Heavy metal association with chronic kidney disease of unknown cause in central India-results from a case-control study

**DOI:** 10.1186/s12882-024-03564-4

**Published:** 2024-04-03

**Authors:** Mahendra Atlani, Ashok Kumar, Rajesh Ahirwar, M. N. Meenu, Sudhir K. Goel, Ravita Kumari, Athira Anirudhan, Saikrishna Vallamshetla, G. Sai Tharun Reddy

**Affiliations:** 1 Department of Nephrology, All India Institute of Medical Sciences (AIIMS), Room No-3022, Academic Block, 3rd Floor, Saket Nagar, Bhopal, Madhya Pradesh 462020 India; 2 Department of Biochemistry, All India Institute of Medical Sciences (AIIMS), Saket Nagar, Bhopal, Madhya Pradesh 462020 India; 3 Department of Environmental Biochemistry, ICMR-National Institute for Research in Environmental Health (NIREH), Bhopal, Madhya Pradesh India; 4 All India Institute of Medical Sciences (AIIMS), Bhopal, Madhya Pradesh India

**Keywords:** Chronic kidney disease of unknown cause, CKDu, Heavy metals, Environmental toxins

## Abstract

**Background:**

Chronic Kidney Disease of unknown cause (CKDu) a disease of exclusion, and remains unexplained in various parts of the world, including India. Previous studies have reported mixed findings about the role of heavy metals or agrochemicals in CKDu. These studies compared CKDu with healthy controls but lacked subjects with CKD as controls. The purpose of this study was to test the hypothesis whether heavy metals, i.e. Arsenic (As), Cadmium (Cd), Lead (Pb), and Chromium (Cr) are associated with CKDu, in central India.

**Methods:**

The study was conducted in a case-control manner at a tertiary care hospital. CKDu cases (*n* = 60) were compared with CKD (*n* = 62) and healthy subjects (*n* = 54). Blood and urine levels of As, Cd, Pb, and Cr were measured by Inductively Coupled Plasma- Optical Emission Spectrometry. Pesticide use, painkillers, smoking, and alcohol addiction were also evaluated. The median blood and urine metal levels were compared among the groups by the Kruskal-Wallis rank sum test.

**Results:**

CKDu had significantly higher pesticide and surface water usage as a source of drinking water. Blood As levels (median, IQR) were significantly higher in CKDu 91.97 (1.3–132.7) µg/L compared to CKD 4.5 (0.0–58.8) µg/L and healthy subjects 39.01 (4.8–67.4) µg/L (*p* < 0.001) On multinominal regression age and sex adjusted blood As was independently associated with CKDu[ OR 1.013 (95%CI 1.003–1.024) *P* < .05].Blood and urinary Cd, Pb, and Cr were higher in CKD compared to CKDu (*p* > .05). Urinary Cd, Pb and Cr were undetectable in healthy subjects and were significantly higher in CKDu and CKD compared to healthy subjects (*P* = < 0.001). There was a significant correlation of Cd, Pb and Cr in blood and urine with each other in CKDu and CKD subjects as compared to healthy subjects. Surface water use also associated with CKDu [OR 3.178 (95%CI 1.029–9.818) *p* < .05).

**Conclusion:**

The study showed an independent association of age and sex adjusted blood As with CKDu in this Indian cohort. Subjects with renal dysfunction (CKDu and CKD) were found to have significantly higher metal burden of Pb, Cd, As, and Cr as compared to healthy controls. CKDu subjects had significantly higher pesticide and surface water usage, which may be the source of differential As exposure in these subjects.

**Supplementary Information:**

The online version contains supplementary material available at 10.1186/s12882-024-03564-4.

## Background

Chronic kidney disease of unknown cause (CKDu) has been reported in various parts of the world (i.e., Nicaragua, El Salvador, Sri Lanka), including India, as an endemic disease. The disease is a diagnosis of exclusion, made when a patient fulfils the Kidney Disease Improving Global Outcomes (KDIGO) CKD criteria but without the evidence of a recognized cause such as diabetes, hypertension, or glomerulonephritis [1]. No uniform and definite cause has yet been identified, though various environmental factors have been associated with and suggested to play a role in the pathogenesis. For example, heat stress, strenuous exercise, agrochemicals, and heavy metals have been held responsible for Mesoamerican nephropathy [2]. Mixed evidence has been reported for association with agrochemicals, heavy metals, and genetic variability for CKDu in Sri Lanka [3–5]. In India, one small study reported an association of heavy metals with CKD [6]. A study done on groundwater samples from the Uddanam region of Andhra Pradesh (India), which has a high prevalence of CKDu reported water as acidic (pH < 6.5) and to contain higher silica and lead in wet and dry seasons, respectively. Phthalates were also detected in the groundwater [7]. Previous studies have attempted to find a correlation between heavy metals and CKDu by comparing cases and endemic and nonendemic controls [4]. No study has yet tried to find the association of heavy metals comparing CKDu with CKD. Furthermore, previous studies have used urinary metal levels as a biomarker of metal exposure. There is an inherent issue of reverse causality i.e., reduced excretion of metals in urine with a reduction in glomerular filtration rate (GFR) [8]. Measurement of metals in blood has also been reported to be a promising biomarker of metal exposure [9, 10]. Some studies have employed urine to blood ratio for deciding whether urine or blood levels should be used for a particular metal. For metals with urine/blood ratio more than one blood metal levels, whereas for metals with urine/blood ratio less than one, urine metal levels were used in estimation analysis [11]. The purpose of this study was to test hypothesis whether heavy metals i.e. Arsenic (As), Cadmium (Cd), Lead (Pb) and Chromium (Cr) are associated with CKDu, in central India using blood and urine levels as biomarker of metal exposure.

## Materials and methods

### Study setting and population

Study was conducted in a tertiary care hospital setting in the Department of Nephrology in India in a case-control design between December 2019 to June 2022. Participants were enrolled between December 2019 -December 2021. The data collection was done simultaneously. The sample analysis was carried out between January to June-2022. The study was performed according to the guidelines of the Declaration of Helsinki. The study objective was to compare CKDu cases with CKD and healthy controls with regard to biomarkers of exposure of heavy metals [blood and urine levels of cadmium (Cd), lead (Pb), arsenic (As) and chromium (Cr)]. The study included adults aged 18–70 years with CKDu and two groups of the control population, one with CKD and another group of healthy controls without evidence of CKD.

The CKDu and CKD cases were inducted among the patients visiting the nephrology outpatient department and based on pre-defined criteria. At the same time, healthy controls were inducted among the healthy relatives accompanying the patients visiting other departments of the institute for treatment. Written informed consent was obtained from all the participants.

The case definition of CKDu was based on criteria proposed by the Indian Society of Nephrology for the diagnosis of CKDu [12]. The inclusion criteria included- eGFR < 60 mL/min/1.73m2 (CKD-EPI) [13] and albumin-to-creatinine ratio (ACR) > 30 mg/g for more than 3 months with:

1. Urine protein creatinine ratio(PCR) less than 2g/g.
2. No history of glomerulonephritis, pyelonephritis, renal calculi, polycystic kidneys or obstruction on renal ultrasound.
3. Not on treatment for diabetes and HbA1c less than 6.5%.
4. Blood pressure less than 140/90 if CKD stage 1 and 2; and less than 160/100 if CKD stage 3,4, and 5 and on a single drug for blood pressure control.

Case definition of CKD was based on: eGFR < 60 mL/min/1.73m2 (CKD-EPI) and albumin-to-creatinine ratio > 30 mg/g for more than 3 months. Patients were included in the CKD group only if PCR > 2g/g. Hypertension with BP > 140/90 in stages 1–2 and > 160/100 in stages 3–5 or on two or more drugs for BP control.

CKD staging was based on the KDIGO-2008 classification [1]. The same stages were applied to categorize the renal functions of subjects with CKDu.

Inclusion criteria for healthy controls included: Absence of CKD as evidenced by eGFR more than 90 ml/min/1.73m2, ACR < 30mg/g and lack of anatomical renal disease, obstruction or stone on renal ultrasound, no history of diabetes, HbA1C less than 6.5 and BP less than 140/90.

Biases were kept a minimum by adhering to the case definition described above, and study exposures are mainly objectively assessed with very less dependency on recall i.e. for pesticide or painkiller use. The urine metal levels were adjusted for urine dilution by estimating metals per gram of creatinine in urine.

### Sample size

Assuming a difference of moderate effect size (0.25), between three groups (CKDu cases, CKD Controls, Normal Controls) with a confidence level of 95% and power of 80%, the calculated sample size was 159. The final sample size estimated, including a 10% non-response rate, was 180 (60 per group).

### Specimen collection and analysis

For the analysis of heavy metals, venous blood (2 ml) was collected in trace element free Trace Element K2-EDTA Vacutainer (Cat# BD 368381). Whole blood was stored at -40 °C until analysis. Ten millilitres (10 ml) of first-morning urine was collected in 50 ml polypropylene tubes. Urine was stored at -40 °C in aliquots until analysis. Serum and urine creatinine was measured using a modified kinetic Jaffe’s method using a Random Access Fully Automated Chemistry Analyzer (Beckman Coulter). Urinary protein and urine albumin were estimated using a colorimetric and immune-turbidimetric methods, respectively, using a Random Access Fully Automated Chemistry Analyzer (Beckman Coulter). HbA1c was analyzed by ion-exchange high pressure liquid chromatography method using a D10 Haemoglobin testing system (BioRad Laboratories). eGFR was calculated from serum creatinine and CKD -EPI equation (Ref). A kidney ultrasound was performed in standard B Mode grey scale in 3.5–5 MHz, the longitudinal length was measured along with the width and thickness of the kidney, renal stones, and any other anatomical abnormality.

### Estimation of heavy metals in blood and urine

Levels of Cd, Pb, Cr and As were measured in whole blood and urine. Urinary spot sample results of metal analysis were adjusted for dilution by urine creatinine. Metal analysis was carried out at NIREH, Bhopal (India).

Levels of various heavy metals, viz. Cd, Pb, Cr, As in the collected blood and urine samples were analyzed through inductively coupled plasma optical emission spectroscopy (iCAP® 7400 Duo ICP-OES, ThermoFisher Scientific® Pvt. Ltd). Blood and urine samples were acid-digested in a microwave oven prior to metal detection on ICP-OES. For blood digestion, 1 mL of whole blood sample was mixed with 6 ml of a freshly prepared mixture of concentrated trace metal grade nitric acid (HNO3) and hydrogen peroxide (H2O2) in a ratio 2:1 (v/v) in high-purity polytetrafluoroethylene (PTFE-TFM) vessels. For urine digestion, 5 mL of urine sample was mixed with 6 ml of a freshly prepared mixture of HNO3 and H2O2 in a ratio of 2:1. After gentle mixing of these reactants with blood, the PTFE-TFM vessels were arranged in the rotor (24HVT80, Anton PAAR) and digestion was carried out in the Anton Paar, multi microwave PRO Reaction System at 200 C for 15 min. Digested samples were cooled to 40°C and diluted to 30 ml with distilled water. Blank was prepared for each cycle of digestion using distilled water, nitric acid, and hydrogen peroxide mixture. All the chemicals were trace-element free.

Before the analysis of metal ions in processed blood and urine samples, calibration standards for each element were prepared from multi-element stock solutions (1000 mg L − 1) in triple distilled water. Detection of Cd, Pb, and Cr was performed using a standard sample introduction setup, whereas for As, the hydride generation sample introduction system was utilized. Online hydride generation for As was achieved with an Enhanced Vapor System sample introduction kit using 0.5% m/v sodium tetrahydroborate (NaBH4) stabilized in 0.5% m/v NaOH and 50% v/v HCl solution. Emission data acquisition was performed using the Qtegra ISDS Software at interference-free wavelengths.

### Statistical analysis

Statistical analyses were performed with R version 4.2 (R Foundation for Statistical Computing, Vienna, Austria) and IBM SPSS 26 version. The distribution of data in groups was evaluated with Shapiro-Wilk, kurtosis, skewness, and histograms. Skewed data for three groups was compared with the Kruskal-Wallis test. Subgroup analysis in three groups was performed with pairwise comparisons by Dunn test. Parameters with homogeneous distribution were compared with the chi-square test. Data are presented as %, for categorical variables or as median (Q1-Q3) for continuous variables.

Detection rates for blood and urinary metal levels were calculated. For urine metal levels, all statistical analyses were performed with creatinine-adjusted metal concentrations.

Urine to blood ratio was calculated for all metal levels. Spearman correlation coefficient was used to find the association between blood and urine metal levels of individual metals as well as for the association between different metals both in blood and urine. Correlation of blood and urine As with GFR was also performed.

We performed multinominal regression analysis for significantly different metal level in CKDu cases with respect to CKD and healthy controls. We included age and gender (confounding factors) in the model to see the y independence of association and effect estimate of the factor associated with CKDu. Regression model matrices and goodness-of-fit were also determined by the pseudo *R*2 coefficient and Hosmer-Lemeshow goodness-of-fit test.

For all analyses, we have considered a *p*-value less than 0.05 as statistically significant.

## Results

A total of 568 patients who visited Nephrology OPD during the study period were screened for inclusion in the study. Out of these, 66 CKDu and 70 CKD cases were found eligible to enroll in the study. Eight patients withdrew consent in the CKD group, whereas four patients in the CKDu group had uncontrolled blood pressure with a single drug, and two withdrew consent. Finally, 60 CKDu and 62 CKD cases were included in the study for outcome analysis. We have approached 120 relatives of patients attending other OPDs and screened them for eligibility criteria of the healthy control group. Out of these, 60 were eligible, and 54 provided consent for participation in the study.

### Demography and lab parameters

The CKD and CKDu subjects were similar in demographics for age and sex. However, healthy subjects were younger (Table 1). There was no significant difference between CKDu and CKD with reference to stage V (32 vs. 44, P-0.107).There were 05 diabetic kidney disease 04 CKD due to secondary glomerular disease patients (3-lupus nephritis, 1-FSGS), 12 hypertension-associated renal disease, 01 ADPKD, 36 Chronic glomerulonephritis patients, and 04 Chronic pyelonephritis patients in the CKD group. Use of smoking, Alcohol, and painkillers was similar across the three groups (Table 1). A significant difference was found between the three study groups with respect to the source of drinking water (ground or surface water). A significantly higher number of CKDu subjects used surface water as a source of drinking water (Table 1 and Table-S1 and Fig-S1) and a higher number of CKDu subjects reported pesticide usage. As shown in Table 1, blood pressures were significantly higher in CKD subjects compared to CKDu and healthy subjects and reflect the inclusion criteria with appropriate patient inclusion in three groups. Both ACR and PCR were also significantly different between CKD and CKDu. The eGFR was calculated based on the CKD-EPI formula and was not significantly different between the CKD and CKDu subjects, however, CKD subjects had lower median eGFR compared to CKDu subjects. The healthy subjects had significantly higher eGFR compared to both groups. HbA1c, were similar across the three groups (Table 1).

**Table 1:** Demography and lab parameters

**Population Characteristics**	CKD, *N* = 62	CKDu, *N* = 60	Healthy, *N* = 54	*P*-value	*P*-value for Pair-wise comparisons by Dunn’s test		
					CKD vs. CKDu	CKD vs. Healthy	CKDu vs. Healthy
Age (Years)	43.0 (32.0, 52.0)	44.0 (36.0, 50.0)	31.0 (27.0, 39.8)	< 0.001*	0.7		
Male	29 (46.8)	26 (43.3)	33 (61.1)	0.136*			
Female	33 (53.2)	34 (56.7)	21 (38.9)				
SBP (mmHg)	142.0 (138.0, 154.0)	138.0 (124.0, 144.0)	125.0 (116.8, 132.0)	< 0.001**	< 0.001	< 0.001	< 0.001
DBP (mmHg)	89.5 (83.0, 97.8)	89.0 (80.0, 94.0)	86.0 (73.2, 89.0)	< 0.001**	0.11	< 0.001	0.015
Smoking (yes)	5 (8.2%)	2 (3.4%)	4 (7.4%)	0.4^#^	0.5	0.7	0.3
Alcohol use (yes)	4 (6.6%)	7 (12%)	3 (5.6%)	0.4^#^	0.3	0.8	0.2
Painkillers use(yes)	1 (1.6%)	6 (10%)	1 (1.9%)	0.08^#^	0.028	> 0.9	0.037
Pesticide use(yes)	7 (12%)	12 (20%)	1 (1.9%)	0.01^#^	0.15	0.1	0.002
Source drinking water (Surface water)	15(24)	25(42)	10(18)	0.016*	0.04	0.458	0.007
ACR1^st^ (mcg/mg)	1,259.4 (487.0, 3,253.6)	321.9 (56.4, 611.6)	13.7 (6.8, 17.4)	< 0.001**	< 0.001	< 0.001	0.1
ACR2^nd^ (mcg/mg)	988.1 (370.5, 2,314.0)	315.8 (98.9, 584.0)	15.0 (8.8, 19.2)	< 0.001**	< 0.001	< 0.001	0.024
PCR (gm/gm)	3.1 (2.0, 5.8)	1.1 (0.4, 1.8)	0.040 (0.020, 0.075)	< 0.001**	0.00	0.00	0.00
HbA1C (pct)	5.4 (5.1, 5.7)	5.4 (5.1, 5.8)	5.8 (5.2, 6.0)	0.12**	> 0.9	0.4	0.12
Sr.Creatinine (mg/dL)	6.1 (3.5, 10.7)	3.8 (1.9, 7.2)	0.8 (0.7, 0.9)	< 0.001**	0.002	< 0.001	< 0.001
eGFR (ml/min/1.73m^2^)	9.0 (6.0, 17.0)	14.5 (7.0, 34.2)	109 (95.0,122.0)	< 0.001**	0.08	0.000	0.000

Median (IQR); n (%) p-values for three group comparison without any superscript was obtained by Kruskal-Wallis rank sum test ** for three group comparison; *Pearson’s Chi-squared test; ^#^Fisher Freeman Halton test CKD-chronic kidney disease, *CKDu* Chronic kidney disease of unknown cause, *SBP* Systolic blood pressure, *DBP* Diastolic blood pressure, *ACR_1st* Albumin creatinine ratio on 1st visit, *ACR_2*^*nd*^ Albumin creatinine ratio on 2^nd^ visit, *Sr* Serum, *eGFR* Estimated glomerular filtration rate, *PCR* Protein creatinine ratio

### Analytical results

The urinary and blood levels of As, Cd, Pb, and Cr (Table 2) were measured in ppb (micrograms per litre), and median with interquartile ranges were reported. Urinary metal levels were also measured in ppb (micrograms per liter) and then adjusted for urinary dilution by urine creatinine value and were finally expressed as micrograms/grams of urine creatinine (Table 2).

**Table 2:** Heavy metal levels comparison in different groups

**Exposures (ppb)**	CKD, *N* = 62	CKDu, *N* = 60	Healthy, *N* = 54	*P* value	*P*-vale for Pair-wise comparisons by Dunn’s test		
					CKD vs. CKDu	CKD vs. Healthy	CKDu vs. Healthy
**As DL (mcg/Lt)**	0.191 ppb						
**U/B ratio**	0.13 (0.00–0.80)	0.73 (0.00–32.37)	2.2 (0.86–70.1)				
**Blood_As (mcg/Lt)**^**a**^	4.5 (0.0, 58.8)	91.97 (1.3, 132.7)	39.01 (4.8, 67.4)	< 0.001*	**0.001**	0.006	0.005
**% above DL**	64.2	78.3	81.2	< 0.001^#^			
**Urine As (mcg/gm)**^**a**^	17.1 (7.7, 29.9)	11.1 (2.8, 22.1)	97.4 (52.4, 152.5)	< 0.001*	0.4	< 0.001	< 0.001
**% above DL**	94	83.4	100	< 0.001^#^			
**Cd DL (mcg/Lt)**	0.0						
**U/B ratio**	0.09 (0.04-.17)	0.10 (0.06–0.21)	0.03 (0.01–0.14)				
**Blood_Cd (mcg/Lt)**^**a**^	10.9 (3.4, 14.0)	9.8 (0.7, 15.2)	2.7 (1.6, 3.8)	< 0.001*	0.5	< 0.001	< 0.001
**% above DL**	91.9	91.6	100	< 0.001^#^			
**Urine_Cd (mcg/gm)**^**a**^	1.0 (0.3, 1.7)	0.5 (0.0, 1.5)	0.0 (0.0, 0.1)	< 0.001*	**0.063**	< 0.001	< 0.001
**% above DL**	80.5	62.8	24.1	< 0.001^#^			
**Lead DL (mcg/Lt)**	0.822						
**U/B ratio**	2.13 (0.38–5.2)	1.6 (0.49–3.47)	0.06 (0.04–0.27)				
**Blood_Pb (mcg/Lt)**^**a**^	54.9 (7.1, 77.6)	27.1 (4.0, 66.3)	10.9 (0.0, 30.0)	< 0.001*	0.14	< 0.001	0.006
**% above DL**	80	85	74.1	0.510^#^			
**Urine_Pb (mcg/gm)**^**a**^	70.5 (17.6, 162.2)	29.0 (1.1, 142.0)	0.0 (0.0, 1.1)	< 0.001*	0.5	< 0.001	< 0.001
**% above DL**	80.6	76.7	29.6	< 0.001^#^			
**Chromium DL (mcg/Lt)**	3.156						
**U/B ratio**	0.13 (0.04–0.18)	0.13 (0.07–0.22)	0.03 (0.01–0.05)				
**Blood Cr (mcg/Lt)**^**a**^	477.2 (253.5, 597.4)	455.0 (156.4, 629.2)	221.4 (177.5, 268.6)	< 0.001*	0.4	< 0.001	< 0.001
**% above DL**	100	86.7	100	< 0.001^#^			
**Urine Cr (mcg/gm)**^**a**^	59.2 (21.7, 82.8)	34.7 (6.3, 82.0)	0.0 (0.0, 0.0)	< 0.001*	0.5	< 0.001	< 0.001
**% above DL**	85.5	86.7	14.8	< 0.001^#^			

^a^Median (IQR); n (%) *p*-values for three group comparison without any superscript was obtained by Kruskal-Wallis rank sum test * for three group comparison. ^#^Pearson’s Chi-squared test. *DL* Detection limit, *U/B* Ratio-urine blood ratio. All values blood metals in ppb (parts per billion or micrograms/liter). All values of urinary metals in microgram/gm). *CKD* Chronic kidney disease, *CKDu* Chronic kidney disease of unknown cause, *As* Arsenic, *Cd* Cadmium, *Pb* Lead, *Cr* Chromium

#### Detection limits

The lowest detectable concentrations of various heavy metals analyzed on ICP OES with a signal-to-noise ratio of 1 were as follows: As (193.759 nm) - 0.191 ppb; Cd (214.438 nm) - 0 ppb; Pb (220.353 nm) - 0.822 ppb; Cr (283.563 nm) - 3.156 ppb (Table 2, Figs-S2-S5).

#### Detection percentage

The number of subjects with blood and urine metal levels above the respective detection limits in each study group is reported in Table 2.

### Urine to blood ratio

A urine/blood ratio for each metal in all study groups was calculated for patients with metal levels above the detection limit. The distribution of urine/blood ratios for all metals is presented in Table 2. Ratios were different between healthy and subjects with deranged kidney functions i.e. low GFR (CKD and CKDu). Median urine/blood Ratio for As was > 1 in healthy subjects and < 1 in CKD and CKDu, reflecting higher urinary levels compared to blood in healthy and reverse in CKD and CKDu subjects. For Pb, it was < 1 in healthy subjects and > 1 in subjects with CKD and CKDu, reflecting higher blood levels compared to urine in healthy and reverse in CKD and CKDu subjects. For Cd and Cr the ratio were < 1 across all three groups suggesting higher urine levels compared to blood levels.

### Correlation

**Fig. 1:**
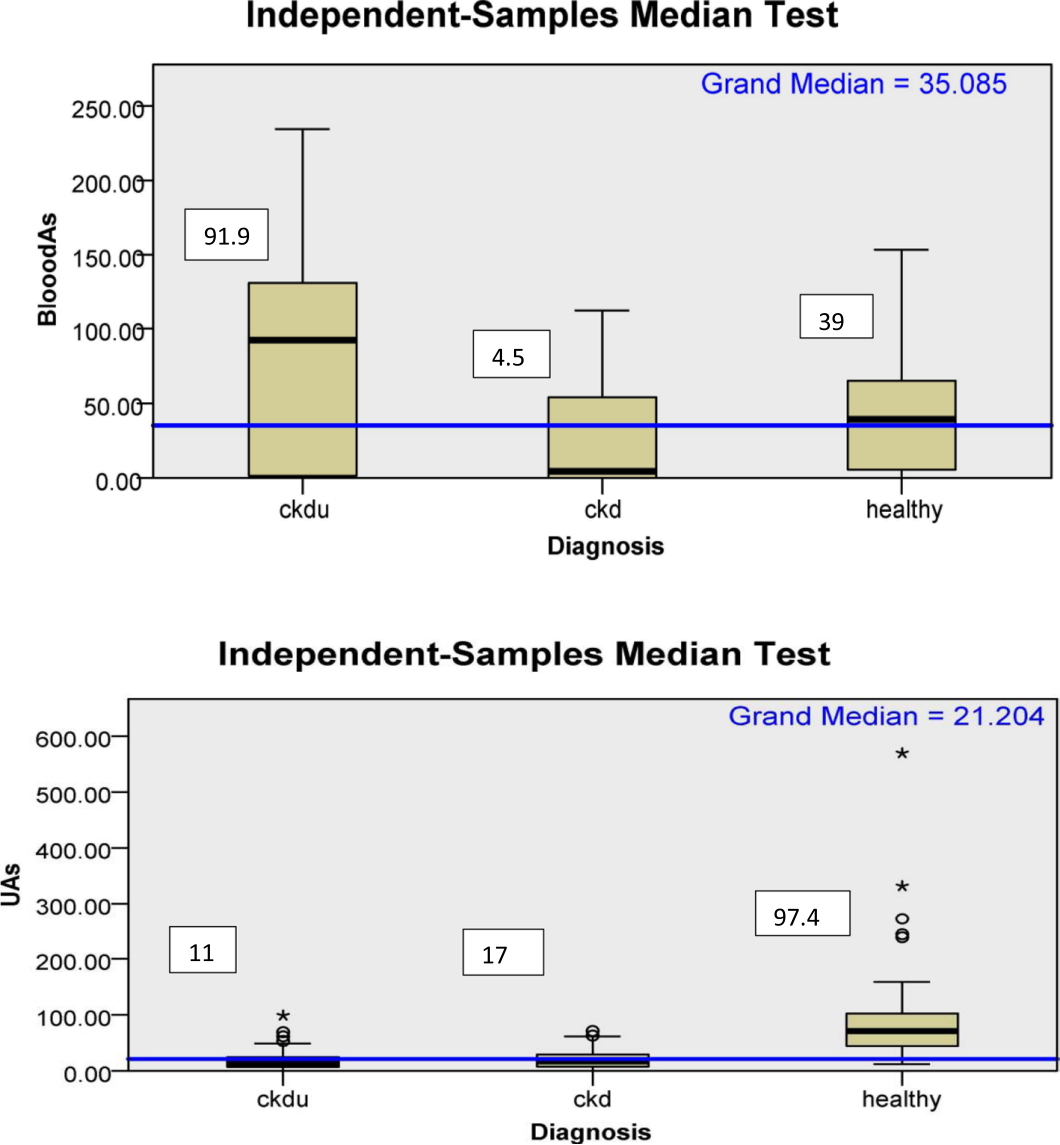
Box plot for distribution of blood and urine arsenic according to diagnosis categories. Median; microgram/Lt (blood); microgram/gm(urine); UAs- Urine arsenic;CKDu-Chronic kidney disease of unknown cause; CKD-Chronic kidney disease

A spearman correlation (ρ) was also performed to see the association between each urine and blood metal and among the metals with each other as well. In CKDu, UAs were negatively associated with BAs (ρ-0.260, *p*-0.11) and in CKD positively (0.138, *p*-0.37). There was a positive association between urine and blood levels of As,Pb, and Cr and negative association of urine and blood Cd in CKD. In CKDu, a positive association was found in blood and urine Cd,Pb and Cr. In addition, there was a strong correlation of blood Cd, Pb, and Cr (*p* < 0.01) [ρ = 0.68 (BCd and BPb), 0.88 (BCd and BCr), 0.71 (BPb and BCr) in CKDu and [ρ = 0.55 (BCd and BPb), 0.82 (BCd and BCr), 0.65 (BPb and BCr) in CKD. The Urine Cd, Pb, and Cr also had strong correlations [ρ = 0.33 (UCd and UPb), and 0.48(UPb and UCr)] in CKD and [ρ = 0.19(UCd and UPb), 0.67 (UCd and UCr), and 0.69 (UPb and UCr)] in CKDu < 0.05 (Table-S2-S4 and Fig-S6). Association of Blood and urine As with GFR was also evaluated, and BAs were found to be negatively associated with GFR (ρ = -0.097, *p* = 0.56), whereas UAs were positively associated (ρ = 0.14, *p* = 0.25) with GFR (Table-S5). **Metal levels: Blood As:** was significantly higher in CKDu (*n* = 37) subjects compared to CKD (*n* = 41) and healthy (*n* = 53) subjects (Table 2). On the other hand, the urinary As (UAs) was significantly low in CKD (*n* = 50) and CKDu (*n* = 48) subjects compared to healthy subjects (*n* = 38) and was non significantly higher in CKD subjects compared to CKDu subjects (Fig. 1, Table 2).The blood and urine As values were below detection limits in 21.6%, 35.7%, and 18.8% and in 6%, 16.6%, and 0% of subjects in CKDu, CKD, and healthy groups, respectively.

#### Cadmium

**Fig. 2:**
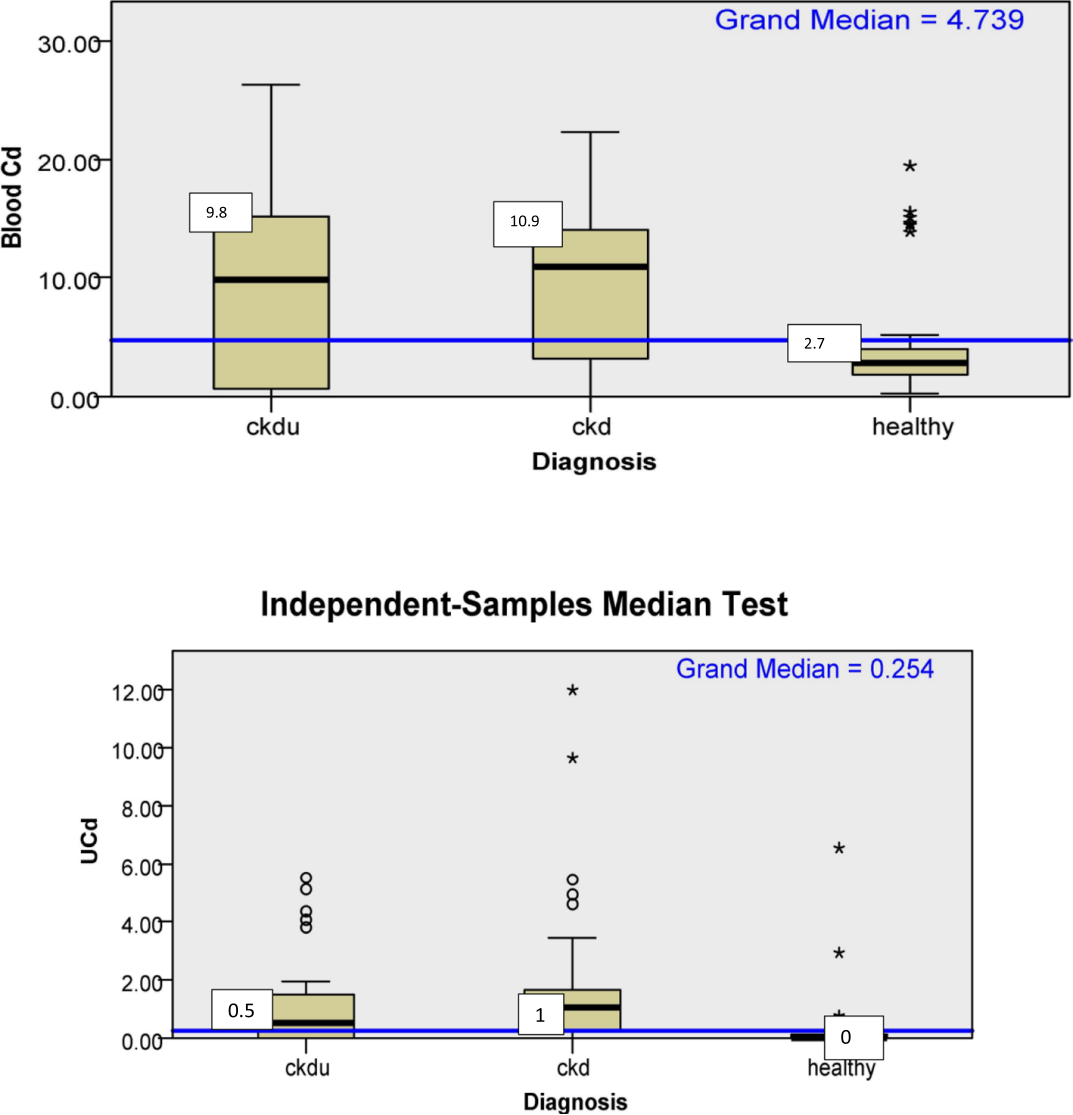
Box plot for distribution of blood and urine cadmium according to diagnosis categories. Median, microgram/Lt (blood); micrograms/gm (urine); UCd- Urine cadmium;CKDu-Chronic kidney disease of unknown cause; CKD-Chronic kidney disease

Blood Cd also was significantly higher in CKD and CKDu subjects compared to healthy subjects. Urinary Cd (UCd) levels were significantly higher in CKD and CKDu subjects compared to healthy subjects,. There was a weak association of (*p* = 0.06) UCd with CKD subjects compared to CKDu subjects.UCd was higher in CKD subjects compared to CKDu (Fig. 2, Table 2). The blood and urine Cd values were below detection limits in 8.3%, 8.1%, and 0% and 37.2%, 19.3%, and 75.9% of subjects in CKDu, CKD, and healthy groups, respectively.

#### Lead

**Fig. 3:**
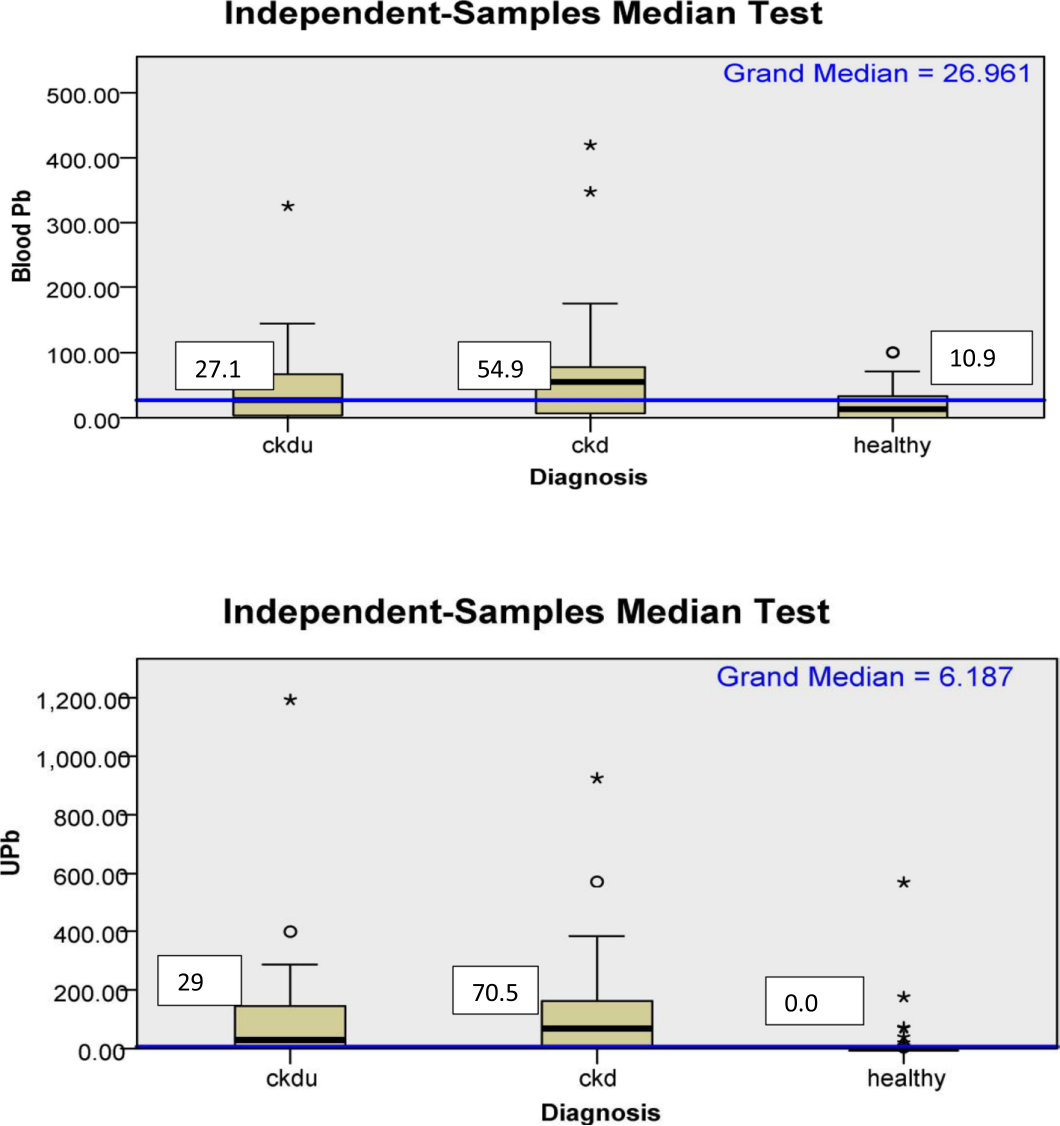
Box plot for distribution of blood and urine lead according to diagnosis categories. microgram/Lt (blood); microgram/gm(urine); UPb- Urine lead;CKDu-Chronic kidney disease of unknown cause; CKD-Chronic kidney disease

Pb levels in the blood of CKD and CKDu as well as in urine of CKD and CKDu subjects were significantly higher compared to healthy subjects. The Pb levels were higher in CKD subjects compared to CKDu subjects, but it was not statistically significant (Fig. 3, Table 2). The blood and urine Pb values were below detection limits in 15%, 20%, and 25.9% and 23.3%, 19.4% and 70.4% of subjects in CKDu, CKD, and healthy groups, respectively.

#### Chromium

**Fig. 4:**
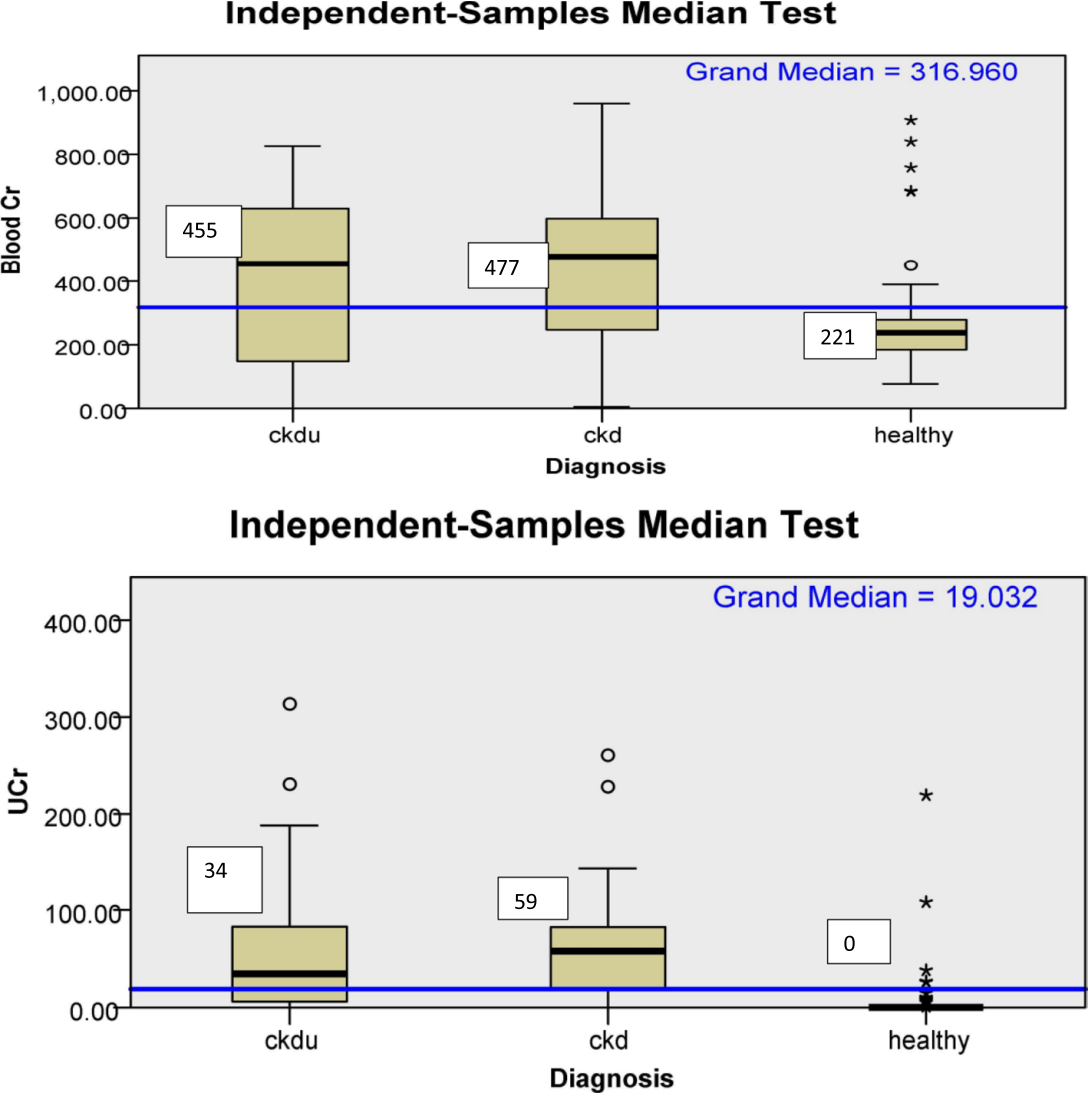
Box plot for distribution of blood and urine chromium according to diagnosis categories. Median; microgram/Lt(blood); microgram/gm(urine); UCr-urine chromium, CKDu-Chronic kidney disease of unknown cause; CKD-Chronic kidney disease

As shown in Table 2 and Fig. 4, urinary and blood Cr was significantly higher in CKD, and CKDu patients than healthy subjects. The blood and urine Cr values were below detection limits in 13%, 0%, and 0% and 13.3%, 14.5% and 85.2% of subjects in CKDu, CKD, and healthy groups, respectively.

### Multinominal regression

Though age, gender, were not significantly different between CKDu and CKD, on univariate analysis, we included these In the multinominal regression analysis between CKDu and CKD in reference to healthy subjects in addition to factors found significantly different (*p* < 0.01 on univariate analysis) i.e. blood As and source of drinking water.After the final model, gender had no association with CKDu. Blood As, surface water as drinking water source and age were independently associated with CKDu. Age was associated independently with CKD also (Table 3).

**Table 3:** Multinominal regression

**Diagnosis**	**Predictors**	**Odds ratio**	**CI**	***P* value**
CKDu	Age	1.104	1.055–1.156	0.000
	Gender	0.964	0.351–2.652	0.944
	Blood As	1.013	1.003–1.024	0.014
	Surface water use	3.178	1.029–9.818	0.045
CKD	Age	1.053	1.013–1.094	0.010
	Gender			0.208
	Blood As	0.988	0.977–1.000	0.041
	Surface water use	1.320	1.320–0.456	0.609
Goodness of fit	Chi-square 0.256			
Pseudo *R*^2^	Cox snell-0.340			

## Discussion

To the best of our knowledge, this is the first study wherein an attempt has been made to analyze the association of heavy metals with CKDu in central India, using blood and urine levels as biomarkers of metal exposure. In addition, CKD and healthy subjects have been used as control groups.

The current study showed that blood and urine creatinine-adjusted urinary levels of heavy metals Cd, Pb and Cr were significantly higher in patients with CKD and CKDu as compared to healthy subjects. The urinary levels of the above metals were undetectable in healthy subjects. The study also showed a weak association of (*p* = < 0.06) higher urinary Cd in CKD subjects compared to CKDu subjects of this Indian cohort.

The study also showed that Blood As was significantly higher in CKDu subjects compared to CKD and healthy subjects. On multinominalregression, blood As was independently (*p* < 0.05) associated with CKDu after age adjustment.

In our study, median GFR was rather high in CKDu subjects [14.5 (7.0, 34.2)] compared to GFR in CKD subjects [9.0 (6.0, 17.0)ml/min/1.73m^2^)] and it was non significantly different between the two groups. On correlation analysis, there was a negative correlation between Blood As and GFR and a positive correlation of urine As with GFR. Based on this, the higher blood As in CKDu with higher GFR appears to be truly elevated.

Previously a study from Sri Lanka has also reported an association of CKDu with chronic As toxicity. In that study, 48% of CKDu patients and 17.4% of the control subjects fulfilled the criteria to be diagnosed with chronic arsenical toxicity(CAT), indicating the potential link between CAT and CKDu and suggesting agrochemicals could be the possible source [14]. Later, it was reported that glyphosate was the most widely used pesticide in Sri Lanka, which contains an average of 1.9 mg/kg arsenic. Findings suggest that agrochemicals, especially phosphate fertilizers, are a major source of inorganic arsenic in CKDu endemic areas [15]. However, another study from Sri Lanka did not find any difference in UAs levels in patients of CKDu in endemic areas and controls from endemic and nonendemic areas [4].

Some other studies have reported associations of As with CKD. A study from Taiwan found total UAs to be associated with a four-fold risk of CKD [6]. Another study reported an association of MMA^V^ (mono methyl arsenate pentavalent) and DMA^V^ (dimethyl arsenate pentavalent) in urine with prevalence of CKD [16]. However, in both studies, the type of CKD was not reported.

The higher blood As in CKDu compared to CKD may be associated with exposures in our study; a significantly higher number of subjects in CKDu group reported use of pesticides, surface water as a source of drinking water in CKDu subjects.On regression analysis also, surface water was independently associated with CKDu.

A study from north India reported increased levels of OCPs, namely α-HCH, aldrin, and β-endosulfan, in CKDu patients as compared to healthy control and CKD patients of known etiology [17] and it is also known that arsenic is an important component of pesticides [18]. The contamination of surface water with various pollutants i.e. pesticides, is common [19]. Arsenic is a known nephrotoxin, and one of the case reports where kidney histopathology was evaluated reported As causes tubulointerstitial disease (TID) [20]. The difference in methylation processes of As has also been found responsible for various diseases associated with As i.e. for example, high proportions of urinary MMAs (%U-MMAs) have been associated with a higher risk of cancers and skin lesions [21]. In contrast, high %U-DMAs has been associated with diabetes risk [22]. We have measured only iAs in our study. Whether methylation resulting in various metabolite species has different associations with CKDu or CKD should be explored further. We recently found a significant association of single nucleotide polymorphism in a gene coding for sodium-dependent dicarboxylate transporter (SLC13A3) with the susceptibility to CKDu [23].

In the current study, the UAs results suggest that As levels of 97 µg/gm of creatinine in healthy subjects were not associated with decreased GFR or proteinuria. Similar results were reported by a study from China where researchers found a lower confidence limit on the benchmark dose (LBMD) of 102 and 0.88 µg/gm creatinine for As and Cd, respectively, in order to prevent renal damage in the general population co-exposed to arsenic and cadmium [24]. The UAs in healthy subjects in our study were nearly similar to the LBMD reference and, not surprisingly, not to be associated with CKD or proteinuria.

Some studies have reported lead to be associated with CKDu. An Indian study reported high levels of lead and silicon concentrations in Indian groundwater in the endemic Uddanam area [7]. Jaysuman et al. also reported higher levels of Pb (26.5 µg/gm) in the urine of patients with Sri Lankan agricultural nephropathy compared to endemic and nonendemic control [25].

In the current study, although the median level of blood Pb was almost double in CKD patients compared to CKDu, the result was not statistically significant.

Our study showed that Cd was significantly associated with renal disease. Blood Cd and urine Cd (UCd) levels were significantly higher in patients with renal disease (CKD and CKDu) as compared to healthy subjects. The findings of UCd also showed a weak association (p-0.06) of Cd with CKD compared to CKDu among patients with renal diseases. There are some concerns that UCd may not be truly reflective of metal burden in patients with advanced CKD [26], because initially, in the course of Cd toxicity with early tubular damage, the normal reabsorption of cadmium-metallothionein decreases, and the UCd concentration increases. However, in the long run, cadmium-induced kidney damage gives rise to low Cd concentrations in both the kidney and urine, while the tubular damage remains [27]. The U/B ratio of < 1 for Cd in our study supports the above findings.

The mean eGFR in our CKD cohort was lower compared to CKDu; despite this, higher UCd values in patients with CKD compared to CKDu in our study indicate a potential association of Cd with CKD.

Studies have reported variable association of Cd with CKDu when compared to healthy subjects. Nanayakkara et al. [28] did not find an association of UCd with CKDu in stages 1–4 compared to healthy controls. Whereas another Sri Lankan [4] study found significantly high UCd in patients with CKDu against the endemic and nonendemic controls. We also observed significantly higher UCd in CKDu vs. healthy controls.

In the current study, urinary Cr (UCr) was not detected in healthy subjects, whereas it was significantly higher in patients with CKD and CKDu as compared to healthy subjects. UCr levels were higher in CKD compared to CKDu. Epidemiologically, Cr exposure has been reported to be associated with kidney damage in occupational populations [26]. Recently, a study from Taiwan reported that a significant and independent association between Cr exposure and decreased renal function in the general population, and co-exposure to Cr with Pb and Cd is potentially associated with an additional decline in the GFR in Taiwanese adults [27]. A study from Bangladesh reported outcomes similar to our study; however, the study included only CKD (*n* = 30) patients and compared them with healthy subjects (*n* = 20). In that study, compared to the controls, CKD patients exhibited significantly higher levels of Pb, Cd, and Cr levels in their urine samples. This signifies a potential association between heavy metal co-exposure and CKD [29]. In the current study a significant correlation between blood Cd, Pb, and Cr and urine Cd, Pb, and Cr were found in CKDu and CKD subjects compared to healthy subjects. The levels of UCd, UPb, and UCr in CKD and CKDu patients were significantly higher compared to healthy controls; The possibility of the combined effect of Cd, Pb, and Cr in the causation of renal diseases could be evaluated further in future studies. As CKDu is an endemic disease, the results of our study suggest an association of arsenic with CKDu in the Indian population, and so the generalizability of the result should be used with caution.

### Strengths and limitations

This is the first study which has included two controls (CKD and healthy) and compared metal levels in patients with CKDu. In addition, the comparison of metals in both blood and urine is another advantage, as falling GFR levels and urine levels of several metals do not reflect true metal burden in patients. Inclusion of CKDu patients, as per the suggested definition by the Indian society of Nephrology, is another strength of our study.

The small sample size of our study may be a limitation of our study though it was calculated scientifically. The study involved Indian patients and controls only so the generalization of the results should be with caution. Healthy controls were of younger age is also a limitation of the study.

Also the study included patients from central India, comparatively a larger area and does not points out endemicity.

## Conclusion

The study finds an association of environmental toxins with CKDu and CKD. The age and sex-adjusted As were observed to have an independent association with CKDu. A weak association of Cd with CKD was also observed in this Indian cohort. Subjects with renal dysfunction (CKDu and CKD) were observed to have a significantly higher metal burden of Pb, Cd, As, and Cr as compared to healthy controls. CKDu patients may have higher exposure to As via pesticides, surface water usage, or both.

### Supplementary Information

**Supplementary Material 1.**

## Data Availability

The datasets used and/or analysed during the current study are available from the corresponding author on reasonable request.

## Abbreviations

CKDu: Chronic Kidney Disease of unknown cause
CKD: Chronic Kidney Disease
As: Arsenic
Cd: Cadmium
Pb: Lead
Cr: Chromium
KDIGO: Kidney Disease Improving Global Outcomes
GFR: Glomerular filteration rate
IHEC: Institutional Human Ethics committee
HNO3: Concentrated trace metal grade nitric acid
H2O2: Hydrogen peroxide
PTFE-TFM: High-purity polytetrafluoroethylene
UAs: Urinary As
UCd: Urinary Cd
UPb: Urine Pb
UCr: Urinary Cr
iAs: Inorganic As
TID: Tubulo-interstitial disease
MMA V: Pentavalent monomethylarsonic acid
DMA V: Pentavalent dimethylarsinic acid
MAs III: Methylarsenous acid
SLC13A3: Sodium-dependent dicarboxylate transporter
LBMD: Limit on the benchmark dose
AsB: Arsenobetaine
U-MMAs: Urinary MMAs
U-DMAs: Urinary DMAs
BLL: Blood lead levels
ESKD: End-stage kidney disease
