# Supplementary material for: Heavy metal association with chronic kidney disease of unknown cause in central India-results from a case-control study
Source: BMC Nephrol. 2024 Apr 3;25:120. doi: 10.1186/s12882-024-03564-4 (PMC10988978; doi:10.1186/s12882-024-03564-4)

| **Supplementary file:**  **Table-S1 Diagnosis * water source group Crosstabulation** | | | | |
| --- | --- | --- | --- | --- |
| Count | | | | |
|  | | water source group | | Total |
|  |  | ground | surface |  |
| Diagnosis | ckdu | 35 | 25 | 60 |
|  | ckd | 47 | 15 | 62 |
|  | healthy | 44 | 10 | 54 |
| Total | | 126 | 50 | 176 |

X^2^- 8.324,df-2 p-.016

Fig-S1: Bar chart comparing drinking water source in CKDu ,CKD, and healthy groups


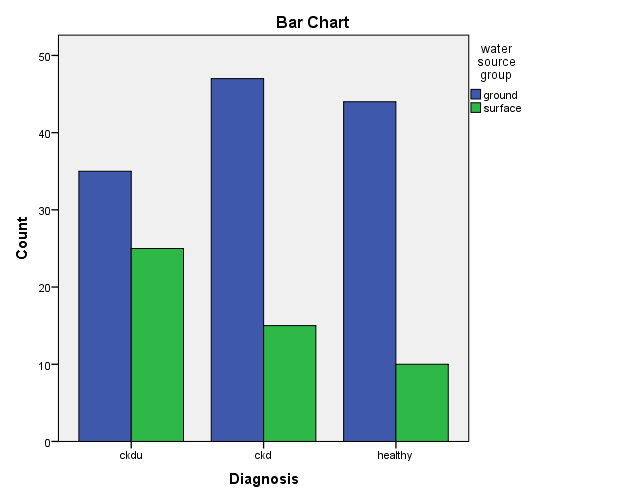


18.5

81.4

24.1

75.8

41.6

58.44

CKDu-Chronic kidney disease of unknown cause, CKD-chronic kidney disease,

|  |
| --- |

Fig-S2: Standard Graphs of analysis for Pb,


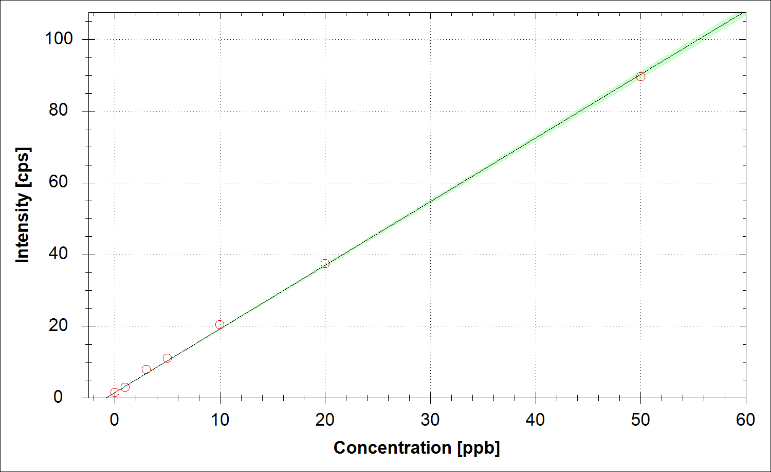


**Pb 220.353 {453} (Axial)**

| f(x) = 1.7746*x + 1.4580 |
| --- |
| R² = 0.9994 |
| BEC = 0.822 ppb |
| LoD = N/A  Fig-S3: Standard Graphs of analysis for Cr, |


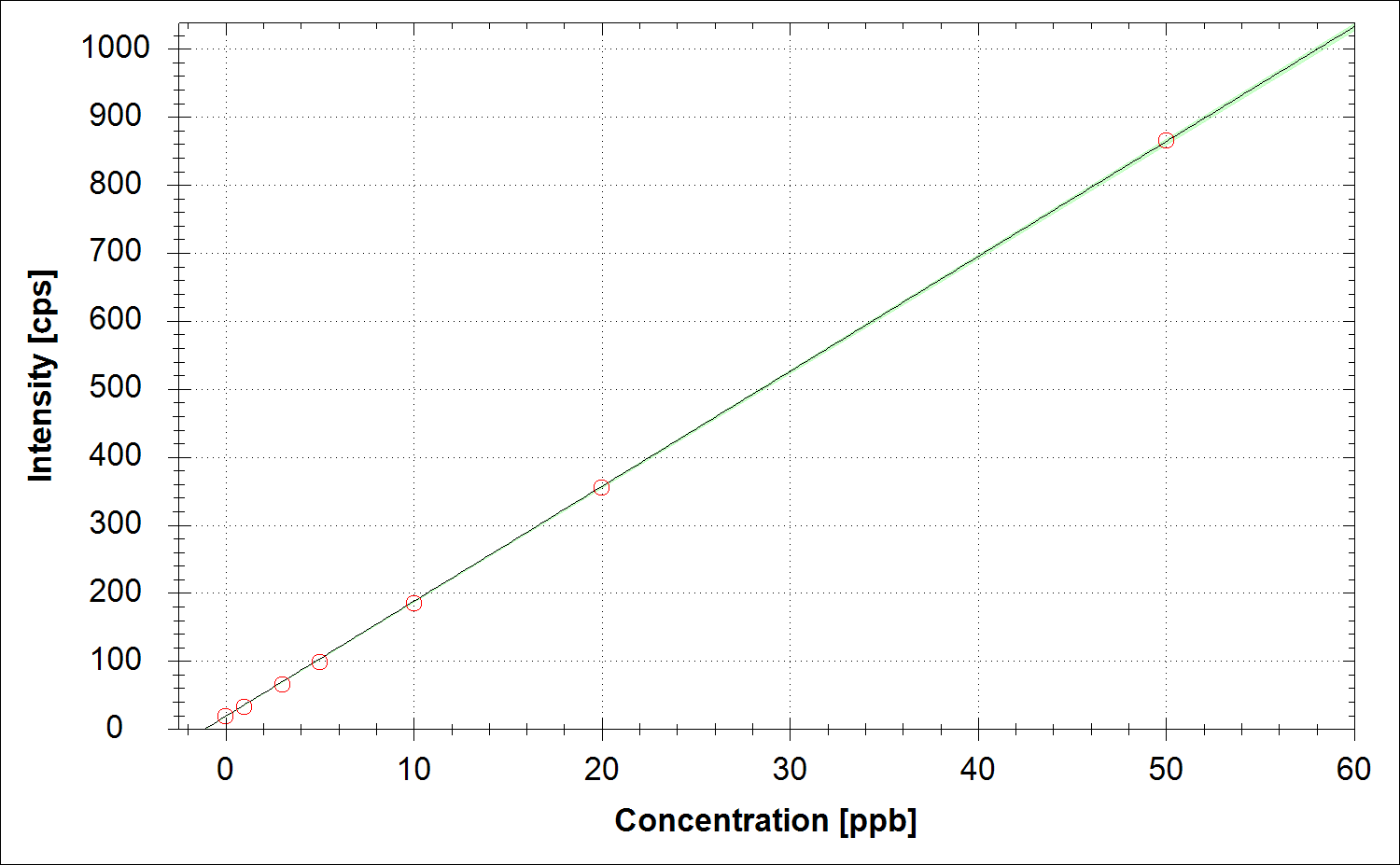


|  |
| --- |
| \| **Cr 283.563 {119} (Axial)** \| \| --- \| \|  \| \| f(x) = 33.1423*x + 104.6082 \| \| R² = 0.9999 \| \| BEC = 3.156 ppb \| \| LoD = N/A \| |
|  |
|  |

|  |
| --- |
|  |

Fig-S4: Standard Graphs of analysis for As,

|  |
| --- |
| \|  \| \| --- \| \| 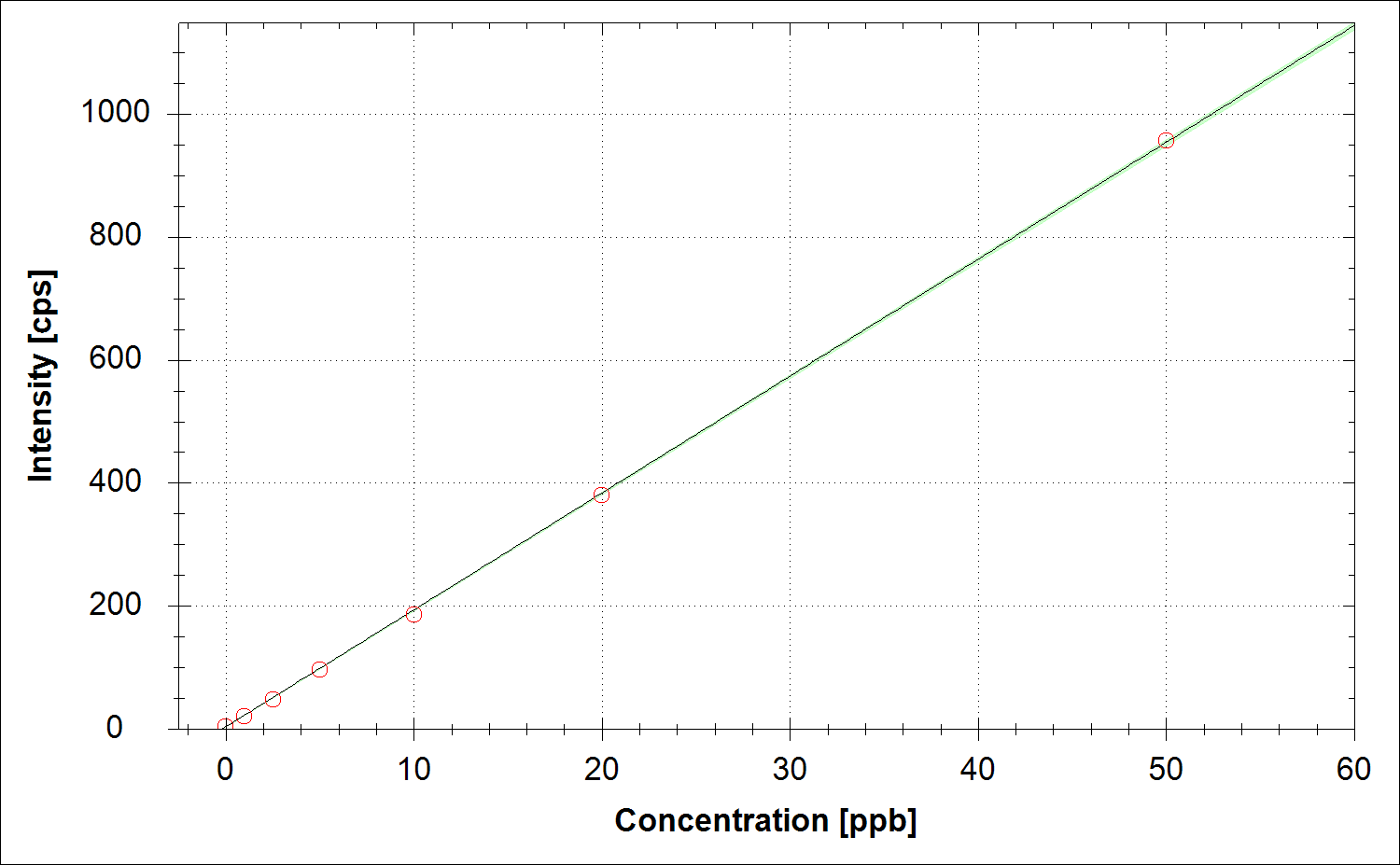 \| \| f(x) = 18.9957*x + 3.6237 \| \| R² = 0.9999 \| \| BEC = 0.191 ppb \| \| LoD = N/A \| \|  \| |

**As 193.759 {474} (Axial)**

**Fig-S5:** Standard Graphs of analysis for Cd,


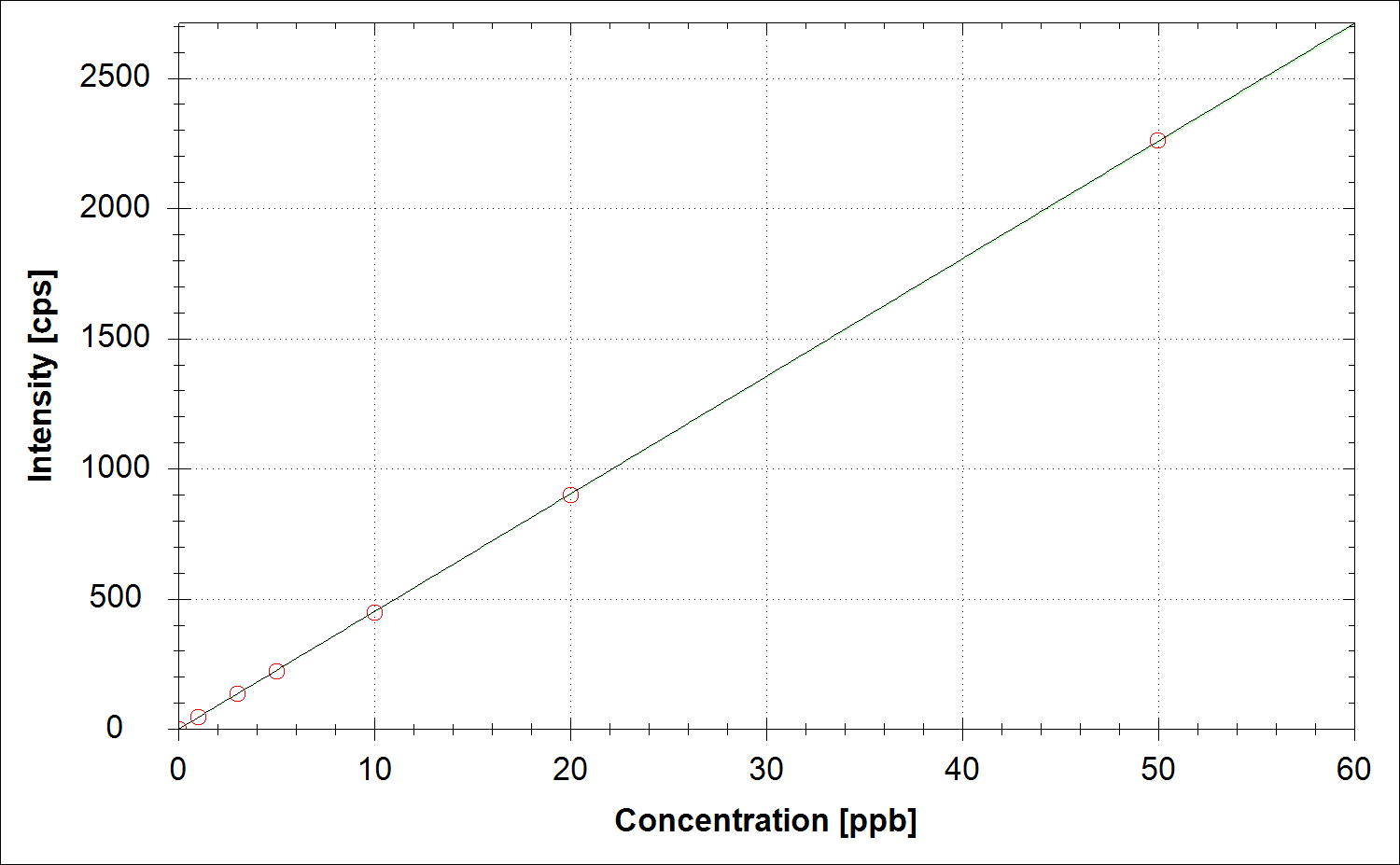


| f(x) = 45.1528*x + -0.0208 |
| --- |
| R² = 1.0000 |
| BEC = 0.000 ppb  **Cd 214.438 {457} (Axial) Standard graphs of analysis** |

| **Table- S2: Correlations of metals in healthy subjects** |
| --- |

| **Table-S-2Correlations of metals in healthy subjects** | | | | | | | | | | |
| --- | --- | --- | --- | --- | --- | --- | --- | --- | --- | --- |
|  | | | BlooodAs | Blood Cd | Blood Pb | Blood Cr | UAs | UCd | UPb | UCr |
| Spearman's rho | BlooodAs | Correlation Coefficient | 1.000 | .294^*^ | .111 | .323^*^ | -.227 | . | .006 | .040 |
|  |  | Sig. (2-tailed) | . | .033 | .427 | .018 | .101 | . | .964 | .779 |
|  |  | N | 53 | 53 | 53 | 53 | 53 | 0 | 53 | 53 |
|  | Blood Cd | Correlation Coefficient | .294^*^ | 1.000 | .386^**^ | .746^**^ | -.370^**^ | -.900^*^ | .275^*^ | .321^*^ |
|  |  | Sig. (2-tailed) | .033 | . | .002 | .000 | .004 | .037 | .034 | .012 |
|  |  | N | 53 | 60 | 60 | 60 | 60 | 5 | 60 | 60 |
|  | Blood Pb | Correlation Coefficient | .111 | .386^**^ | 1.000 | .366^**^ | -.281^*^ | -.200 | .273^*^ | .139 |
|  |  | Sig. (2-tailed) | .427 | .002 | . | .004 | .029 | .747 | .035 | .291 |
|  |  | N | 53 | 60 | 60 | 60 | 60 | 5 | 60 | 60 |
|  | Blood Cr | Correlation Coefficient | .323^*^ | .746^**^ | .366^**^ | 1.000 | -.462^**^ | -.400 | .218 | .307^*^ |
|  |  | Sig. (2-tailed) | .018 | .000 | .004 | . | .000 | .505 | .095 | .017 |
|  |  | N | 53 | 60 | 60 | 60 | 60 | 5 | 60 | 60 |
|  | UAs | Correlation Coefficient | -.227 | -.370^**^ | -.281^*^ | -.462^**^ | 1.000 | .100 | -.172 | -.162 |
|  |  | Sig. (2-tailed) | .101 | .004 | .029 | .000 | . | .873 | .188 | .216 |
|  |  | N | 53 | 60 | 60 | 60 | 60 | 5 | 60 | 60 |
|  | UCd | Correlation Coefficient | . | -.900^*^ | -.200 | -.400 | .100 | 1.000 | 1.000^**^ | .900^*^ |
|  |  | Sig. (2-tailed) | . | .037 | .747 | .505 | .873 | . | . | .037 |
|  |  | N | 0 | 5 | 5 | 5 | 5 | 5 | 5 | 5 |
|  | UPb | Correlation Coefficient | .006 | .275^*^ | .273^*^ | .218 | -.172 | 1.000^**^ | 1.000 | .656^**^ |
|  |  | Sig. (2-tailed) | .964 | .034 | .035 | .095 | .188 | . | . | .000 |
|  |  | N | 53 | 60 | 60 | 60 | 60 | 5 | 60 | 60 |
|  | UCr | Correlation Coefficient | .040 | .321^*^ | .139 | .307^*^ | -.162 | .900^*^ | .656^**^ | 1.000 |
|  |  | Sig. (2-tailed) | .779 | .012 | .291 | .017 | .216 | .037 | .000 | . |
|  |  | N | 53 | 60 | 60 | 60 | 60 | 5 | 60 | 60 |
| *. Correlation is significant at the 0.05 level (2-tailed). | | | | | | | | | | |
| **. Correlation is significant at the 0.01 level (2-tailed). | | | | | | | | | | |

| **Table-S3 Correlations Metals in CKDu** | | | | | | | | | | | |
| --- | --- | --- | --- | --- | --- | --- | --- | --- | --- | --- | --- |
|  | | | BlooodAs | Blood Cd | Blood Pb | Blood Cr | UAs | UCd | UPb | UCr |  |
| Spearman's rho | BlooodAs | Correlation Coefficient | 1.000 | -.567^**^ | -.529^**^ | -.483^**^ | -.260 | -.149 | -.640^**^ | -.505^**^ |  |
|  |  | Sig. (2-tailed) | . | .000 | .001 | .002 | .114 | .626 | .000 | .001 |  |
|  |  | N | 38 | 38 | 38 | 38 | 38 | 13 | 38 | 38 |  |
|  | Blood Cd | Correlation Coefficient | -.567^**^ | 1.000 | .687^**^ | .882^**^ | .227 | .266 | .736^**^ | .513^**^ |  |
|  |  | Sig. (2-tailed) | .000 | . | .000 | .000 | .078 | .140 | .000 | .000 |  |
|  |  | N | 38 | 61 | 61 | 61 | 61 | 32 | 61 | 61 |  |
|  | Blood Pb | Correlation Coefficient | -.529^**^ | .687^**^ | 1.000 | .711^**^ | -.076 | -.150 | .510^**^ | .340^**^ |  |
|  |  | Sig. (2-tailed) | .001 | .000 | . | .000 | .561 | .413 | .000 | .007 |  |
|  |  | N | 38 | 61 | 61 | 61 | 61 | 32 | 61 | 61 |  |
|  | Blood Cr | Correlation Coefficient | -.483^**^ | .882^**^ | .711^**^ | 1.000 | .155 | .270 | .634^**^ | .495^**^ |  |
|  |  | Sig. (2-tailed) | .002 | .000 | .000 | . | .232 | .135 | .000 | .000 |  |
|  |  | N | 38 | 61 | 61 | 61 | 61 | 32 | 61 | 61 |  |
|  | UAs | Correlation Coefficient | -.260 | .227 | -.076 | .155 | 1.000 | .471^**^ | .290^*^ | .366^**^ |  |
|  |  | Sig. (2-tailed) | .114 | .078 | .561 | .232 | . | .006 | .023 | .004 |  |
|  |  | N | 38 | 61 | 61 | 61 | 61 | 32 | 61 | 61 |  |
|  | UCd | Correlation Coefficient | -.149 | .266 | -.150 | .270 | .471^**^ | 1.000 | .198 | .678^**^ |  |
|  |  | Sig. (2-tailed) | .626 | .140 | .413 | .135 | .006 | . | .277 | .000 |  |
|  |  | N | 13 | 32 | 32 | 32 | 32 | 32 | 32 | 32 |  |
|  | UPb | Correlation Coefficient | -.640^**^ | .736^**^ | .510^**^ | .634^**^ | .290^*^ | .198 | 1.000 | .693^**^ |  |
|  |  | Sig. (2-tailed) | .000 | .000 | .000 | .000 | .023 | .277 | . | .000 |  |
|  |  | N | 38 | 61 | 61 | 61 | 61 | 32 | 61 | 61 |  |
|  | UCr | Correlation Coefficient | -.505^**^ | .513^**^ | .340^**^ | .495^**^ | .366^**^ | .678^**^ | .693^**^ | 1.000 |  |
|  |  | Sig. (2-tailed) | .001 | .000 | .007 | .000 | .004 | .000 | .000 | . |  |
|  |  | N | 38 | 61 | 61 | 61 | 61 | 32 | 61 | 61 |  |
| **. Correlation is significant at the 0.01 level (2-tailed). | | | | | | | | | | | |
| *. Correlation is significant at the 0.05 level (2-tailed). | | | | | | | | | | | |

| **Table-S 4 Correlations of Metals in CKD patients** | | | | | | | | | | |
| --- | --- | --- | --- | --- | --- | --- | --- | --- | --- | --- |
|  | | | BlooodAs | Blood Cd | Blood Pb | Blood Cr | UAs | UCd | UPb | UCr |
| Spearman's rho | BlooodAs | Correlation Coefficient | 1.000 | -.529^**^ | -.449^**^ | -.449^**^ | .138 | -.466^*^ | -.443^**^ | -.185 |
|  |  | Sig. (2-tailed) | . | .000 | .003 | .003 | .378 | .019 | .003 | .235 |
|  |  | N | 43 | 43 | 43 | 43 | 43 | 25 | 43 | 43 |
|  | Blood Cd | Correlation Coefficient | -.529^**^ | 1.000 | .558^**^ | .820^**^ | -.164 | -.047 | .590^**^ | .356^**^ |
|  |  | Sig. (2-tailed) | .000 | . | .000 | .000 | .211 | .777 | .000 | .005 |
|  |  | N | 43 | 60 | 60 | 60 | 60 | 39 | 60 | 60 |
|  | Blood Pb | Correlation Coefficient | -.449^**^ | .558^**^ | 1.000 | .656^**^ | .029 | .027 | .328^*^ | .410^**^ |
|  |  | Sig. (2-tailed) | .003 | .000 | . | .000 | .826 | .869 | .011 | .001 |
|  |  | N | 43 | 60 | 60 | 60 | 60 | 39 | 60 | 60 |
|  | Blood Cr | Correlation Coefficient | -.449^**^ | .820^**^ | .656^**^ | 1.000 | .025 | .083 | .509^**^ | .219 |
|  |  | Sig. (2-tailed) | .003 | .000 | .000 | . | .850 | .615 | .000 | .093 |
|  |  | N | 43 | 60 | 60 | 60 | 60 | 39 | 60 | 60 |
|  | UAs | Correlation Coefficient | .138 | -.164 | .029 | .025 | 1.000 | .547^**^ | .011 | .093 |
|  |  | Sig. (2-tailed) | .378 | .211 | .826 | .850 | . | .000 | .934 | .479 |
|  |  | N | 43 | 60 | 60 | 60 | 60 | 39 | 60 | 60 |
|  | UCd | Correlation Coefficient | -.466^*^ | -.047 | .027 | .083 | .547^**^ | 1.000 | .338^*^ | .067 |
|  |  | Sig. (2-tailed) | .019 | .777 | .869 | .615 | .000 | . | .035 | .687 |
|  |  | N | 25 | 39 | 39 | 39 | 39 | 39 | 39 | 39 |
|  | UPb | Correlation Coefficient | -.443^**^ | .590^**^ | .328^*^ | .509^**^ | .011 | .338^*^ | 1.000 | .483^**^ |
|  |  | Sig. (2-tailed) | .003 | .000 | .011 | .000 | .934 | .035 | . | .000 |
|  |  | N | 43 | 60 | 60 | 60 | 60 | 39 | 60 | 60 |
|  | UCr | Correlation Coefficient | -.185 | .356^**^ | .410^**^ | .219 | .093 | .067 | .483^**^ | 1.000 |
|  |  | Sig. (2-tailed) | .235 | .005 | .001 | .093 | .479 | .687 | .000 | . |
|  |  | N | 43 | 60 | 60 | 60 | 60 | 39 | 60 | 60 |
| **. Correlation is significant at the 0.01 level (2-tailed). | | | | | | | | | | |
| *. Correlation is significant at the 0.05 level (2-tailed). | | | | | | | | | | |

| **Table-S-5 Correlations of blood and urine arsenic with GFR** | | | | | |
| --- | --- | --- | --- | --- | --- |
|  | | | BlooodAs | UAs | GFR |
| Spearman's rho | BlooodAs | Correlation Coefficient | 1.000 | -.260 | -.097 |
|  |  | Sig. (2-tailed) | . | .114 | .564 |
|  |  | N | 38 | 38 | 38 |
|  | UAs | Correlation Coefficient | -.260 | 1.000 | .148 |
|  |  | Sig. (2-tailed) | .114 | . | .256 |
|  |  | N | 38 | 61 | 61 |
|  | GFR | Correlation Coefficient | -.097 | .148 | 1.000 |
|  |  | Sig. (2-tailed) | .564 | .256 | . |
|  |  | N | 38 | 61 | 61 |

Fig-S6-Heat map of metal correlations


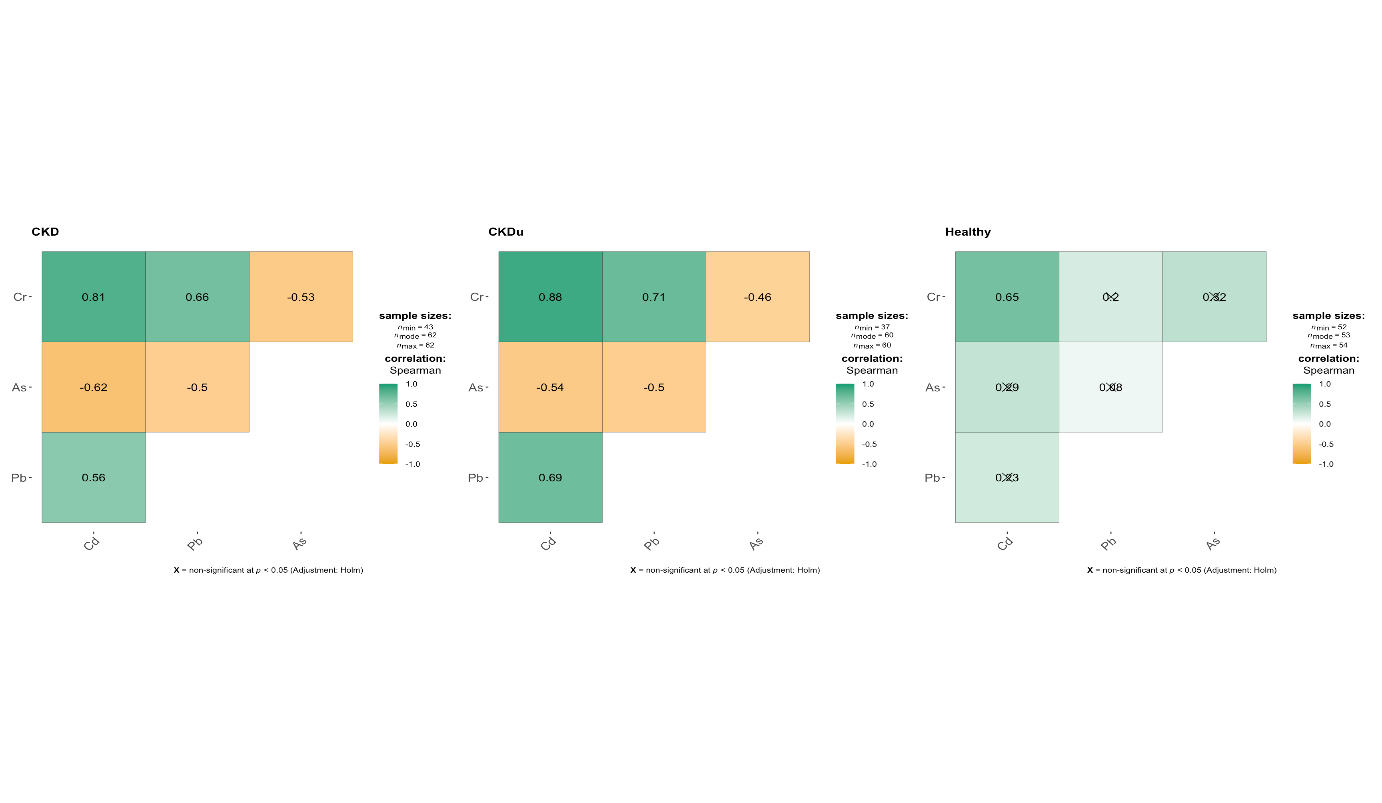

Supplement: Supplementary file 1 — Supplementary Material 1. [file 12882_2024_3564_MOESM1_ESM.docx]
